# Transvesicoscopic Repair of Vesicovaginal Fistula

**DOI:** 10.1155/2010/760348

**Published:** 2010-02-10

**Authors:** R. B. Nerli, Mallikarjun Reddy

**Affiliations:** Department of Urology, KLES Kidney Foundation, KLES Dr. Prabhakar Kore Hospital, Belgaum 590010, India

## Abstract

*Introduction*. Vesicovaginal fistula has been a social and surgical problem for centuries. Many surgical techniques have been developed to correct this abnormality, including transabdominal, transvaginal, and endoscopic approaches. The best approach is probably the one with which the surgeon feels most experienced and comfortable. Laparoscopy has become increasingly popular in urology, reducing the invasiveness of treatment and shortening the period of convalescence. We report our results of transvesicoscopic approach for VVF repair. *Materials and Methods*. Patients with VVF were offered repair using the transvesicoscopic route. With the patient under general anaesthesia and in modified lithotomy position cystoscopy was performed with gas insufflation. Under cystoscopic guidance the bladder was fixed to anterior abdominal wall and ports inserted into the bladder. The fistula was repaired under endoscopic vision. *Results*. Four women, who had VVF following abdominal hysterectomy, underwent this procedure. The operating time ranged from 175 to 235 minutes. There was minimal bleeding. Post operative complications included ileus in one and fever in another. No recurrence of VVF was noted in any patient. *Conclusions*. Transvesicoscopic repair of VVF is feasible, safe, and results in lower morbidity and quicker recovery time.

## 1. Introduction

Vesicovaginal fistulas present a debilitating and stressful condition for women in all parts of the world. Surgical repair remains the primary method of treatment, regardless of the etiology. Controversies still exist regarding the timing and surgical approach of Vesicovaginal fistulas repair. The goal of treatment of Vesicovaginal fistula (VVF) is the rapid cessation of urine leakage with return of normal and complete urinary and genital function. It has been stated that the best opportunity to achieve successful repair of VVF is with the initial operation [1]. Previous failed attempts at repair produce scar and anatomic distortion and may compromise potential reconstructive flaps. There is no best approach for all patients with VVF. Classically VVF has been repaired through a transvaginal or transabdominal approach. Each approach has merits, depending on the particular circumstances of the fistula, and excellent outcomes can be expected with both approaches [2]. Laparoscopic approaches to VVF have been reported [3–5]. Surgeons using laparoscopic approach claim several advantages of laparoscopic repair such as shorter hospital stay, more rapid postoperative recovery; and better cosmetic results than the traditional abdominal approach. Laparoscopy allows an excellent view and good exposure of pelvic structures and provides quick and direct access to the fistula, and relatively simple fistula resection [6]. We report our series of VVF treated by transvesicoscopic approach.

## 2. Materials and Methods

Patients with VVF formed the study group. A detailed history and examination was done in all patients. A three-swab test was done to confirm the clinical suspicion. A routine ultrasonography of kidney, ureters, and bladder region was done in all. Imaging studies included Cystograms, Intravenous urogram, Magnetic resonance imaging when felt necessary and appropriate. Cystoscopy was done to identify the fistula and note its size, position, and sorroundings. At the same instance vaginoscopy was done to observe the end of the fistula. VVF repair was performed at least 12 weeks after its occurrence. 

### 2.1. Surgical Technique

**Figure 1:**
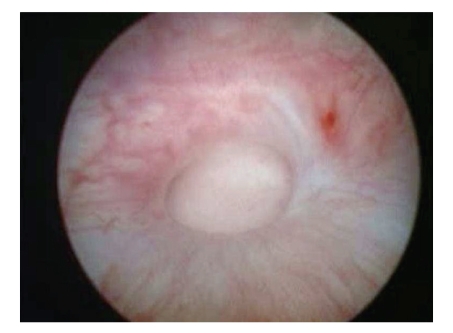
Transvesicoscpic vision of VVF.

The patient was placed in modified lithotomy position. An initial cystoscopy was performed using insufflation of gas and the fistula inspected in detail (Figure 1). The bladder was fixed to the anterior abdominal wall under cystoscopic guidance. The bladder was fixed using 1/0 prolene and placed by using a technique of looping the suture material into the bladder with the help of a spinal needle and then hooking it with the same suture through a neighbouring site.

**Figure 2:**
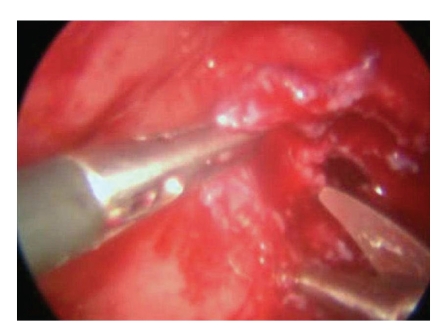
Circumfistula dissection being made.

**Figure 3:**
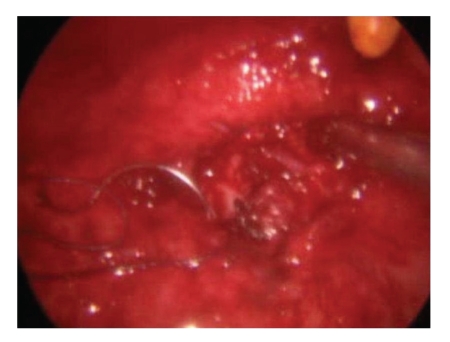
Closure of vagina vertically.

**Figure 4:**
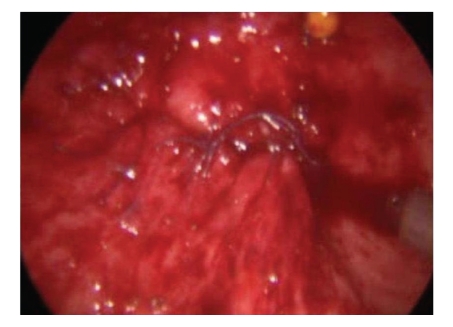
Bladder closure completed.

A 5 mm endoscopic port was placed into the bladder under cystoscopic guidance in the midline, halfway between the umbilicus and pubic symphysis. Two more working/instrument ports were placed 5 cm laterally and inferior to the endoscopic port on either side. Once the ports were in place the cystoscope was withdrawn and the urethra catheterised. The vagina was packed with betadine packs so as to prevent gas leak. The fistula was once again inspected. A circum fistula incision was made and the bladder dissected away from the underlying vagina (Figure 2). The edges of the fistula were excised. Once adequate dissection was achieved, the vagina was sutured vertically (Figure 3) and the bladder edges sutured horizontally (Figure 4). The bladder was closed using 4/0 vicryl. The two ureteric orifices were catheterised using 5F infant feeding tubes and brought outside the bladder for drainage. The bladder was also catheterised. The infant feeding tubes were removed after one week and the catheter removed after two weeks. 

All patients underwent three-swab test, on table cystograms and cystoscopy in the follow-up period. All patients were reassessed six months after surgery. They all were requested to answer a questionnaire related to their act of micturition, satisfaction with the outcome of their surgery, and sexual performance.

## 3. Results

Four patients with history of VVF following gynaecologic surgical procedure presented to us for repair during the period Jan. 2008–Jan. 2009. Their ages ranged from 42 years to 58 years. All the four had undergone total abdominal hysterectomy. All the fistulas were located superior to the trigone, away from the ureteric orifices. The size of the fistulas ranged from 1 cm to 2 cm. 

None of these patients had undergone previous repairs. Transvesicoscopic laparoscopy was performed in all the three patients under general anaesthesia and in modified lithotomy position. Operative time ranged from 175 minutes to 235 minutes. The first case was the one with the maximum time taken. Conversion to open was not done in any patient. Blood loss was minimal in all. Intraoperative difficulties were noted in the first case, which included fixing the bladder to the anterior abdominal wall, pressure of insufflation to maintain the pneumovesicum during port insertion and suction, suturing of the vagina and bladder with continuous urine pool, and lastly the small space for instrument movement. These difficulties were less in the second and thereafter cases as we were able to overcome these initial discomforts. 

In the immediate postoperative period no obvious complications were noted, one patient developed upper respiratory tract infection and fever which subsided on its own. Another patient developed prolonged ileus more than 24 hours, which again settled on its own. Three patients were started orally within 24 hours and the patient with ileus was started orally after 48 hours. Patients were allowed to move within 36 hours. All patients had their infant feeding tubes removed on the 7th postoperative day and discharged with urethral catheters. Catheters were removed after 15 days following surgery. Follow-up ranges from 8 months to 17 months in these patients. No recurrence of VVF was noted in any one of them. 

All the four patients were satisfied with the surgical outcome; voiding was near normal in all. The two patients who were sexually active prior to surgery, continued to be having sexual relationship though both experienced some discomfort initially.

## 4. Discussion

There is no “best” approach for all patients with VVF. Although factors such as size, location, and need for adjunctive procedures often have an impact on the choice of approach, the most important factor is commonly the experience of the operating surgeon [2]. Thus, there is no preferred approach for all fistulas, and the optimal approach to the uncomplicated postgynecologic VVF is usually the one that is most successful in the individual surgeon's hands [7]. Although it has been a long-held belief that gynaecologists prefer to fix VVF transvaginally and urologists prefer a transabdominal approach because of their respective training and experience [8, 9], this difference is becoming increasingly blurred as urologists gain more experience and comfort operating transvaginally for a number of different indications. 

The majority of VVF's are amenable to a transvaginal repair. The relative advantages of a transvaginal approach compared with an abdominal approach include shorter operating times, briefer hospital stay, and less blood loss [10]. The principal disadvantages of the transvaginal approach include the relative lack of familiarity of the vaginal cuff anatomy to many urologists; the potential for vaginal shortening, especially with the Latzko approach; the difficulty in exposing high or retracted fistulas near the vaginal cuff, especially in deep, narrow vaginas or those without any apical prolapse, such as that found in nulliparous women [2]. VVF may be repaired transabdominally, and this is the preferred approach in those cases requiring augmentation cystoplasty or ureteral reimplantation. Compared with the vaginal approach, the transabdominal approach to VVF repair is associated with longer recovery time and inpatient hospitalization, greater blood loss, more cosmetic deformity, and in general, greater morbidity [2]. 

Minimally invasive approaches to VVF repair would be ideal. A number of case reports and small series of laparoscopic approach have already been published [3–6]. C. H. Nezhat et al. [11] first reported laparoscopic repair of a VVF. Von Theobald et al. [3] used an omental J-flap interposition during the laparoscopic repair of VVF. Recurrent VVF was similarly successfully repaired laparoscopically by Miklos et al. [4]. Their patient had previous two failed Latzko partial colpoclesis and closing the vagina and bladder with an interposed omental flap using a laparoscopic approach ultimately repaired the persistent fistula. Similar success with laparoscopic approach was described by a number of other authors [5, 12, 13]. The various authors were of the opinion that laparoscopy offered the patient several advantages which included shorter hospital stay, more rapid postoperative recovery, and better cosmetic appearance than the traditional abdominal approach. The long operative time (>300 minutes) was attributed to difficulty in identification of the fistulous tract, difficult dissection of the Vesicovaginal space, and need for intracorporeal suturing [6]. Sotelo et al. [14] incorporated concomitant cystoscopy to help guide the bladder incision, facilitating quick access to the VVF, and avoiding unnecessary dissection in the Vesicovaginal space. 

With our past experience in laparoscopy and intracorporeal suturing we decided on attempting transvesicoscopic approach. Several laparoscopic surgeons have used this approach in a number of situations such as transvesicoscopic reimplantation of ureters, extraction of huge vesical calculi, and prostatectomy. All our four patients had a solitary, supratrigonal VVF away from ureteric orifices. Transvesicoscopic approach led us directly over the fistula, making dissection of bladder from vagina easy. The vision was good and the intracorporeal suturing easy.

Transvesicoscopic repair of VVF carries all the advantages of laparoscopy including minimal invasiveness, less morbidity, shorter hospital stay, early recovery, and better cosmetic appearance. The disadvantages of standard transabdominal laparoscopy, such as injury to other intraperitoneal organs, need for peritoneal drain, prolonged ileus, bleeding, are avoided in this technique. One obvious disadvantage of this procedure would be the inability to interpose healthy tissue such as omentum, in between the bladder and vagina. But with improved experience, articulated instruments, and newer devices, surgeons in future may be able to develop Martius flap and interpose in between the vagina and bladder.

## 5. Conclusions

Transvesicoscopic repair of a Vesicovaginal fistula is feasible, safe and provides good results. It is an additional modification to the laparoscopic transabdominal approach with all the advantages of laparoscopy.
